# Measurement invariance of the parent-reported Strengths and Difficulties Questionnaire in autistic adolescents

**DOI:** 10.1177/13623613241236805

**Published:** 2024-03-13

**Authors:** Chloe Turcan, Henry Delamain, Asher Loke, Richard Pender, Will Mandy, Rob Saunders

**Affiliations:** University College London, UK

**Keywords:** adolescents, autism spectrum disorders, psychiatric comorbidity, quality of life, screening

## Abstract

Mental health conditions are more prevalent in autistic people than non-autistic people. The Strengths and Difficulties Questionnaire is a commonly used screening tool for emotional and behavioural difficulties in autistic children and adolescents. However, few studies have assessed the psychometric properties of the Strengths and Difficulties Questionnaire in populations of autistic people, especially measurement invariance, to examine whether the underlying latent structure is consistent across time or groups. Measurement invariance of the parent-reported Strengths and Difficulties Questionnaire in a nationally representative cohort study was examined: longitudinal invariance (autistic 11-, 14- and 17-year-olds) and group invariance (autistic and non-autistic 17-year-olds). Differential item functioning was explored in the autistic/non-autistic group analysis. The five-factor structure showed inadequate fit, especially in the autistic group; by extension, measurement invariance could not be established. Differential item functioning was observed for several conduct problems, prosocial behaviour and peer problems subscale items, as well as reverse-coded items. Poor structural validity and item-level invariance may confound findings from research within and between populations of autistic and non-autistic adolescents, as well as screening of mental health conditions in autistic adolescents. This research calls for the development and improvement of screening tools for emotional and behavioural difficulties in populations of autistic people while accounting for their heterogeneity.

**Lay abstract:**

Autistic people are more likely than non-autistic people to experience mental health difficulties. The Strengths and Difficulties Questionnaire is often used to screen for these difficulties and to otherwise make important decisions about mental health treatment and research in populations of autistic people. However, this study suggests that parent-reported Strengths and Difficulties Questionnaire scores may not be useful for comparing autistic and non-autistic adolescents at 11, 14 and 17 years old, as well as screening for mental health conditions in autistic adolescents. In addition, several items may be more likely to be endorsed by parents of autistic 17-year-olds than by parents of non-autistic 17-year-olds (and vice versa), which might suggest caution is needed when comparing groups on specific items.

## Introduction

Autism spectrum disorder (hereafter ‘autism’) is a neurodevelopmental condition characterized by difficulties in social communication, as well as restricted, repetitive behaviours and interests (American Psychiatric Association, 2013), with a prevalence of around 1.5% (Lyall et al., 2017). A range of mental health and neurodevelopmental conditions are more common in autistic people than the general population, with 70% to 95% of autistic children and adolescents, as well as 73% to 81% of autistic adults, being estimated to meet the criteria for at least one condition (Mosner et al., 2019). For example, M. C. Lai et al.’s (2019a) meta-analysis estimated the prevalence of attention-deficit hyperactivity disorder (ADHD) and anxiety disorders at 28% and 20% in autistic people compared to 7.2% and 7.3% in non-autistic people, respectively, with similar findings for depressive, conduct, sleep–wake and other disorders. They highlighted the need for screening, assessment and treatment that accounts for the comorbidity between autism and mental health conditions, rather than addressing these diagnoses separately. Mental health conditions in autistic people persist from childhood through adolescence (Simonoff et al., 2013) and adulthood (Joshi et al., 2013), contributing to negative outcomes such as additional impairments in social adjustment beyond the difficulties in social communication associated with the core autism phenotype (Chiang & Gau, 2016). Greater prevalence of mental health conditions is also associated with lower quality of life across various domains (i.e. physical health, psychological, social relationships and environment; Mason et al., 2018) and higher rates of premature mortality (e.g. via higher suicide risk; Hirvikoski et al., 2016).

Addressing mental health conditions in populations of autistic people is a key priority for improving quality of life (McConachie et al., 2020), as reports by autistic adults (Jones et al., 2014) and parents of autistic children (Crane et al., 2016) suggest that the autism diagnostic process and post-diagnostic support are inadequate. For example, clinicians’ stereotyped beliefs and lack of specialized knowledge, as well as the rigidity of service systems, may worsen or fail to address mental health conditions in autistic people (Brede et al., 2022). Longitudinal research is also needed to understand risk factors and developmental pathways of mental health conditions in this population (Rubenstein & Bishop-Fitzpatrick, 2019). The use of valid screening tools for mental health conditions is key to pursuing this line of research and supporting clinicians to identify specific needs for this population. For example, the Strengths and Difficulties Questionnaire (SDQ; Goodman, 1997) is an emotional and behavioural questionnaire for children and adolescents assessing emotional symptoms, conduct problems, hyperactivity/inattention, peer problems and prosocial behaviour. The SDQ is frequently used to screen for emotional and behavioural difficulties in children and adolescents with neurodevelopmental disorders (Grasso et al., 2022), as well as to evaluate mental health interventions for autistic adolescents by measuring changes in mean total difficulties scores over time (e.g. Shochet et al., 2022). While the SDQ has been used to make clinically relevant decisions about mental health conditions in autistic people, its psychometric properties have not been studied extensively in this population.

In non-autistic populations, previous research has shown mixed findings for the reliability and validity of the SDQ. Given that Cronbach’s α has been argued to underestimate reliability and to poorly reflect internal structure (Sijtsma, 2009), alternative coefficients which account for item ordinality and non-normal distribution, such McDonald’s ω, have been recommended (Revelle & Zinbarg, 2009) and used to assess the reliability of the SDQ. Stone et al. (2015) found acceptable reliability of the parent- and teacher-reported total difficulties scores and subscale scores across 4- to 7-year-olds (ω ⩾ .70). However, concerns have been noted at the subscale level, especially the parent-reported conduct problems and peer problems subscales across 4- to 10-year-olds (ω < .70; Ribeiro Santiago et al., 2022) and most self-reported total difficulties subscales across 12- to 16-year-olds (especially conduct problems; Kankaanpää et al., 2023). Unreliability at the subscale level increases risk of misclassification when using total difficulties scores to screen for mental health conditions (Kankaanpää et al., 2023). For example, based on Charter and Feldt’s (2001) findings on the effects of unreliability on clinical decisions, Ribeiro Santiago et al. (2022) estimated that, for a reliability coefficient of ω = .65, 40% of true positive cases of mental health conditions in Australian 4- to 11-year-olds would be misclassified. Furthermore, issues with test–retest reliability have been reported, especially for the parent-reported compared to teacher-reported SDQ (*r* < .70 for parent-reported subscales; Stone et al., 2010); however, weak correlations between scores over time could reflect true changes in emotional and behavioural difficulties, rather than unreliable measurement. Overall, these findings from studies of 4- to 17-year-olds suggest that further research is needed to understand the psychometric properties of the SDQ, especially at the subscale level.

Few studies have addressed the psychometric properties of the SDQ in populations of autistic people. Similar to non-autistic populations, the self- and parent-reported SDQ shows moderate-to-good validity: inter-rater reliability (*r* = .42) comparable to non-autistic samples (*r* = .48), as well as strong associations with other measures of emotional symptom- and hyperactivity/inattention-related disorders (i.e. good external validity; Findon et al., 2016). Meanwhile, Murphy et al. (2018) suggested that the parent-reported SDQ may be a valid screening tool for disorders related to emotional symptoms (e.g. anxiety disorders) and hyperactivity/inattention (e.g. ADHD) in autistic people, as the emotional and hyperactivity/inattention subscales correlated with other screening and diagnostic measures for these disorders (i.e. good external validity) and showed high sensitivity in predicting disorder risk (e.g. detected 90% of emotional disorder cases). Few studies have assessed the reliability of the SDQ in populations of autistic people using recommended coefficients such as McDonald’s ω. Vugteveen et al. (2020) found acceptable reliability of the parent-reported SDQ in a clinical sample of 12- to 17-year-olds (ω ⩾ .80 except for peer problems), with relatively weaker reliability for the self-reported conduct problems (ω = .65) and peer problems (ω = .69) subscales. However, participants were drawn from primary care data and did not contain exclusively autistic participants.

Moreover, while psychometric properties like reliability and external validity have been studied extensively in the SDQ, they may be impacted by instrumental bias. For instance, as previously mentioned, weaker test–retest reliability for the parent-reported SDQ (Stone et al., 2010) may be attributable to either true score changes or unreliable measurement. Measurement invariance (MI), which has been overlooked in many previous studies, represents the extent to which an instrument measures the same construct across time or groups, allowing for the possibility of unreliable measurement to be assessed (van de Schoot et al., 2012). An invariant instrument suggests that the same people at different time points or people from different groups interpret the measure in the same way, and that the latent structure is the same across these groups. MI is necessary to draw meaningful conclusions from longitudinal or group comparisons. Conversely, if MI does not hold, the same people at different time points or people from different groups may interpret the measure differently. As such, true differences may be confounded by methodological artefacts (e.g. unintended measurement of secondary latent constructs), and meaningful conclusions from score comparisons are more difficult to make (Millsap & Kwok, 2004).

MI of the parent-reported SDQ has been examined both longitudinally and at the group level (e.g. between genders) in 3- to 17-year-olds (Murray et al., 2022). However, some subgroups (e.g. 17-year-old males) showed poor model fit, demonstrating the importance of assessing MI when using the SDQ to make score comparisons. These findings were replicated for gender and neighbourhood deprivation invariance by Staatz et al. (2021), who noted that cross-loadings between subscales may be responsible for non-invariance of specific items in the emotional symptoms, conduct problems and prosocial behaviour subscales. Similarly, Vugteveen et al. (2021) found that the five-factor model – where items load onto their respective subscales – showed poorer fit in clinical samples compared to community samples.

Despite the frequent use of the SDQ in research and practice, previous studies have not investigated MI in populations of autistic people. Establishing longitudinal invariance would allow changes over time to be measured and linked to interventions, while group invariance would ensure that meaningful comparisons can be made between autistic and non-autistic populations.

Therefore, this study aimed to (1) examine longitudinal invariance of the parent-reported SDQ for autistic 11-, 14- and 17-year-olds and (2) assess group invariance between autistic and non-autistic 17-year-olds. Differential item functioning (DIF) complemented the second aim by examining non-invariance of individual items between groups – that is, to identify which specific items apply differently to the autistic and non-autistic groups and require further investigation to understand whether these items are unfairly biased towards either group.

## Methods

### Participants

Participants (*N* = 19244 pre-exclusion criteria) were parents of cohort members (CMs) from the Millennium Cohort Study (MCS; Connelly & Platt, 2014): a nationally representative birth cohort study following UK-based participants from birth through adolescence. Data at each sweep were collected in-person, online and via post. Data from the parent-reported SDQ were used when CMs were around 11 years old (Sweep 5), 14 years old (Sweep 6) and 17 years old (Sweep 7). CMs who left the study before Sweep 5 were excluded, as were those who were missing any SDQ data at either Sweep 5, 6 or 7.

### Measures

The parent-reported SDQ (Goodman, 1997) is a screening questionnaire for young people aged 2 to 17 years, comprising 25 items divided into five subscales containing five items each: emotional symptoms, conduct problems, hyperactivity/inattention, peer problems and prosocial behaviour. Responses are recorded on a 3-point Likert-type scale: ‘Not True (0)’; ‘Somewhat True (1)’; ‘Certainly True (2)’. Items 7, 11, 14, 21 and 25 were reverse-coded. Subscale scores were calculated by summing scores for all items on each subscale, and total difficulties scores by summing all subscales except prosocial behaviour.

Sex was determined based on Sweep 3 or 4 reports. Ethnicity and banded family income – combining total net income for lone parents and couples (Rosenberg et al., 2020) – were determined based on Sweep 1.

Autism diagnosis was determined based on a binary variable collected at Sweeps 3, 4, 5 and 6, which asks parents whether CMs were diagnosed with ‘autism, Asperger’s syndrome or other ASD’ by a medical professional. CMs were recorded as autistic or non-autistic based on the latest available information, ignoring missing data. For example, a CM coded as autistic at Sweep 5 followed by missing data at Sweep 6 was recorded as autistic, while a CM coded as autistic followed by non-autistic at a later sweep was recorded as non-autistic; alternatively, participants coded as autistic at Sweep 7, but as non-autistic or missing data at previous sweeps, were recorded as autistic.

### Data analysis

#### MI testing

**Figure 1.**
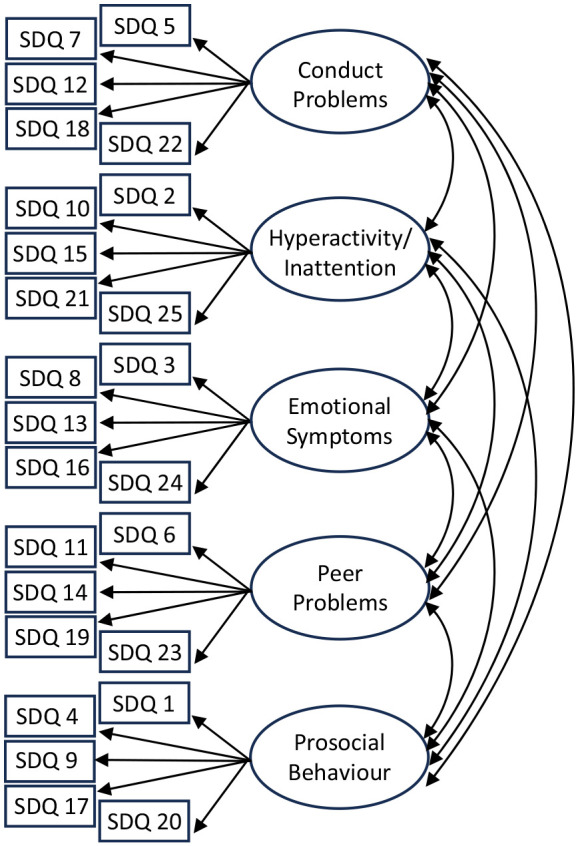
Five-factor model. Circles represent latent constructs, while rectangles represent manifest variables. Single arrows show factor loadings, while double-headed arrows show covariances. SDQ = Strengths and Difficulties Questionnaire.

Multiple-group confirmatory factor analysis (MG-CFA) was used for MI testing. MG-CFA consists of modelling relationships between manifest variables and latent constructs – in this case, items and subscales. We used the originally proposed five-factor model (Figure 1), which has shown good fit for the parent-reported SDQ in a non-autistic sample (Goodman, 2001). MI was tested between groups to assess whether the SDQ measures the same construct across these groups: sweep (three levels: 11-, 14- and 17-year-olds) for the longitudinal analysis; autism diagnosis (two levels: autistic and non-autistic) for the group analysis. For the longitudinal analysis, the autistic group was analyzed independently to compare results to previous findings of longitudinal invariance in non-autistic populations (Murray et al., 2022). For the group analysis, 17-year-olds were analyzed independently as this was the most recent sweep for which autism diagnosis information was available, allowing for adolescents with a later age of diagnosis to be identified.

First, a single-group CFA model was fitted for each group (i.e. 11-, 14- and 17-year-olds for the longitudinal analysis or autistic and non-autistic for the group analysis) without parameter constraints to test for deviations from the five-factor structure. If model fit was adequate (discussed below) for all groups, a series of hierarchical tests was conducted using MG-CFA. For each test, if changes in model fit from one level to the next were acceptable, an additional constraint was imposed across groups: factor structure at the configural level; factor loadings at the metric level; intercepts at the scalar level; residual variances at the residual level. Configural non-invariance suggests differences in factor structure between groups. Metric non-invariance and scalar non-invariance suggest that item responses and mean score differences, respectively, differ due to instrumental bias. Highly constrained levels of MI like residual invariance are difficult to achieve in practice, as this level represents a situation in which latent constructs are measured identically (i.e. with the same amount of error) across groups. However, only scalar invariance must hold for groups’ latent mean scores to be meaningfully compared (van de Schoot et al., 2012).

Following Sass, Schmitt and Marsh’s (2014) recommendations, different estimation methods for single- and multi-group CFA were compared to assess the stability of the results. Maximum likelihood (ML), the default estimator for *lavaan* (Rosseel, 2012), treats data as continuous. Meanwhile, the weighted least squares mean- and variance-adjusted (WLSMV) estimator from *lavaan* (Rosseel, 2012) tends to perform better with ordinal data and with less than five response categories compared to ML estimation (Sass et al., 2014); scaled chi-square test results and fit indices were reported for this estimator. While WLSMV was more theoretically justifiable for the SDQ, as the items can be considered ordinal with three response categories, the single-group CFA model for autistic 11-year-olds failed to converge. This was most likely due to smaller sample size in the autistic group, and as a result, to specific items having few observations for specific response categories (e.g. three observations for Item 21 in the 11-year-old autistic group). As such, results from ML estimation were reported and, where possible, compared to results from WLSMV estimation (see Supplemental Appendix B for models fit with WLSMV estimation).

Hu and Bentler’s (1999) criteria were used to assess model fit using comparative fit index (CFI), Tucker–Lewis index (TLI), root mean square error of approximation (RMSEA) and standardized root mean square residual (SRMR). Fit was considered good if CFI and TLI were ⩾.95; it was considered good if RMSEA and SRMR were ⩽.06, adequate if 0.6 < RMSEA and SRMR ⩽ 0.8 and inadequate if RMSEA and SRMR were >.08. Based on Sass et al.’s (2014) recommendations, the following criteria were used to assess changes in model fit: significant chi-square test, ΔCFI ⩽ –.002, ΔTLI ≠ 0 (Marsh et al., 2010) and ΔRMSEA ⩾ .007 (Meade et al., 2008) suggest non-invariance, as well as ΔSRMR ⩾ .025 for configural/metric levels and ΔSRMR ⩾ .005 for scalar/residual levels (for small or unequal sample sizes; Chen, 2007). However, Sass et al. (2014) highlighted the need to rely on the chi-square test and interpret changes in fit indices with caution when using WLSMV estimation.

In cases of non-invariance, partial invariance can be considered (Meitinger et al., 2020); for example, if fit is inadequate at the scalar level, partial scalar invariance may be established if potential causes of non-invariance (e.g. non-invariant items) are identified through alternative statistical measures (e.g. DIF).

#### Differential item functioning

DIF analysis identifies items to which groups respond differently due to instrumental bias, which threatens the validity of group comparisons. Martinková et al.’s (2017) DIF analysis methods were used: the Mantel–Haenszel chi-square test (Mantel & Haenszel, 1959), which identifies items as non-DIF if the odds of responding to the item are similar across groups (i.e. odds ratio αMH around 1). DIF items favouring the reference (i.e. non-autistic) group had αMH >1; those favouring the focal (i.e. autistic) group had αMH <1. The standard metric delta scale (ΔMH) indicated effect size:|ΔMH| < 1 was negligible;|ΔMH| ⩾ 1.5 was large; 1 ⩽ |ΔMH| < 1.5 was moderate. The Benjamini–-Hochberg *p* value correction was used to control Type I error rate while maximizing power (Kim & Oshima, 2013).

Data analysis was conducted in R (R Core Team, 2022) using *lavaan* (Rosseel, 2012) and *difR* (Magis et al., 2015).

### Community involvement statement

The research team included practitioners with experience working with autistic people and their families, including therapeutic support and co-production of mental health services and training.

## Results

### Descriptive statistics

**Figure 2.**
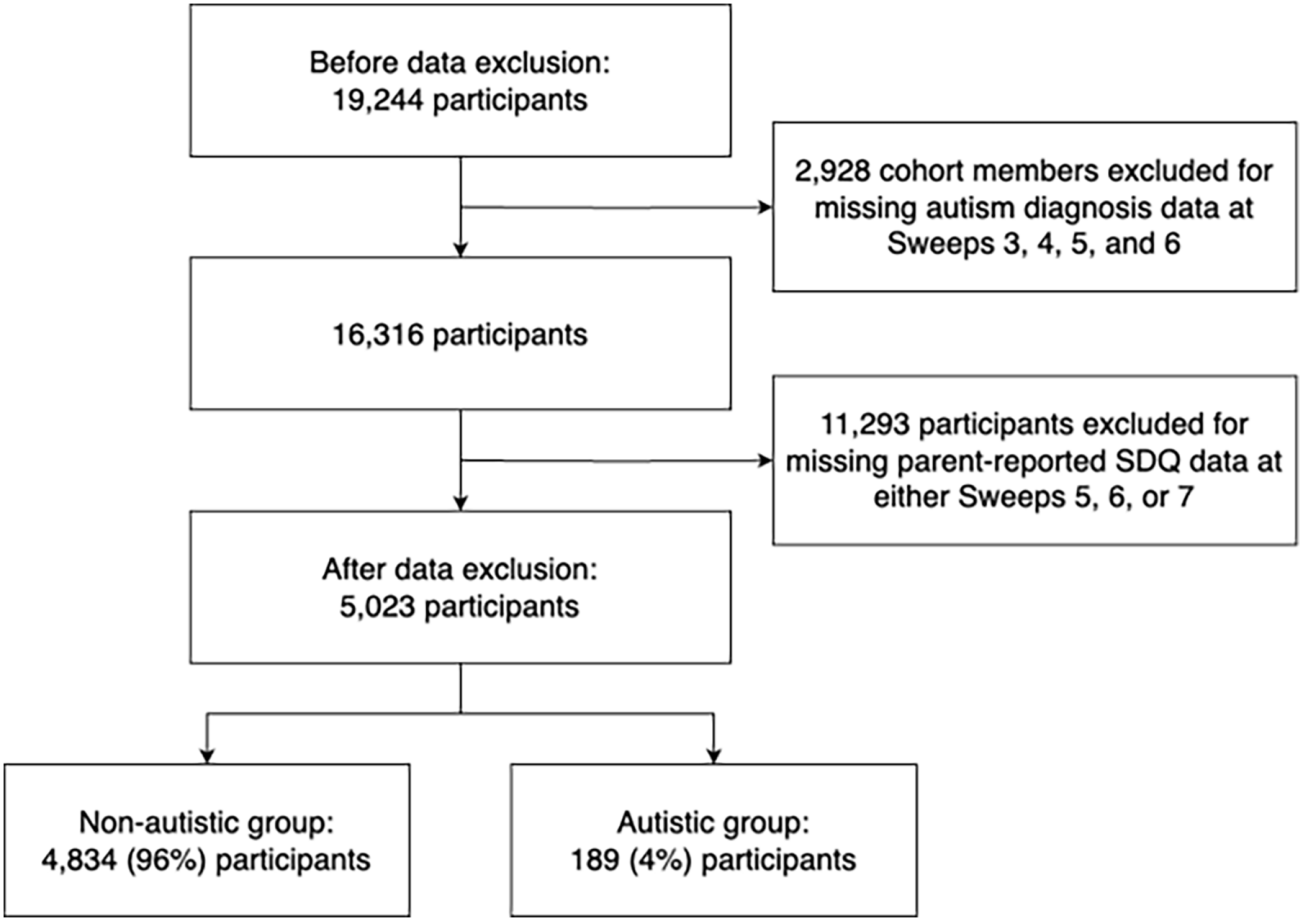
Exclusion criteria. SDQ = Strengths and Difficulties Questionnaire.

A total of 5023 participants had autism diagnosis data available for at least one sweep and parent-reported SDQ data available across all sweeps (Figure 2). A total of 4834 (96%) CMs were non-autistic, while 189 (4%) CMs were autistic. Table 1 contains demographic information.

**Table 1.** Demographic information and descriptive statistics.

Variable	Response	Non-autistic	Autistic		
*N*		4834 (96%)	189 (4%)		
Sex					
	Female	2518 (52%)	46 (24%)		
	Male	2248 (47%)	138 (73%)		
	Missing	68 (1%)	5 (3%)		
Ethnicity					
	White	4196 (86.80%)	165 (87%)		
	Missing	148 (3.06%)	9 (5%)		
	Mixed	123 (2.54%)	10 (5%)		
	Pakistani	120 (2.48%)	0		
	Indian	80 (1.65%)	0		
	Black African	51 (1.06%)	1 (1%)		
	Bangladeshi	39 (0.081%)	0		
	Black Caribbean	30 (0.062%)	3 (2%)		
	Other ethnic group	18 (0.037%)	0		
	Other Asian	17 (0.035%)	1 (1%)		
	Other Black	8 (0.017%)	0		
	Chinese	4 (0.008%)	0		
Family income					
	£55000+ pa	367 (7.59%)	9 (4.76%)		
	£33000–£55000 pa	1185 (24.51%)	34 (17.99%)		
	£22000–£33000 pa	990 (20.48%)	40 (21.16%)		
	£11000–£22000 pa	1174 (24.29%)	55 (29.10%)		
	£3300–£11000 pa	524 (10.84%)	34 (17.99%)		
	£0–£33000 pa	153 (3.17%)	1 (0.053%)		
	Missing	441 (9.12%)	16 (8.47%)		
Total difficulties scores					
		*n*	*M*	*SD*	Range
Age 11					
	Autistic	189	18.38	4.82	8–31
	Non-autistic	4834	12.62	3.35	2–31
Age 14					
	Autistic	189	17.95	7.58	0–38
	Non-autistic	4834	6.98	5.20	0–32
Age 17					
	Autistic	189	17.70	4.98	8–36
	Non-autistic	4834	12.75	3.54	4–33
Prosocial behaviour scores					
		*n*	*M*	*SD*	Range
Age 11					
	Autistic	189	7.07	2.33	0–10
	Non-autistic	4834	8.98	1.36	0–10
Age 14					
	Autistic	189	6.62	2.33	0–10
	Non-autistic	4834	8.51	1.71	0–10
Age 17					
	Autistic	189	6.74	2.25	0–10
	Non-autistic	4834	8.63	1.66	0–10

Percentages were given within groups (e.g. 73% of autistic cohort members were male).

On average, across all sweeps, parents in the autistic group reported higher total difficulties scores (*M* = 18.01, *SD* = 5.92) than the non-autistic group (*M* = 10.78, *SD* = 4.92), *t*(596.89) = –28.66, *p* < 0.001. Parents in the autistic group reported lower prosocial behaviour scores (*M* = 6.81, *SD* = 2.31) than the non-autistic group (*M* = 8.71, *SD* = 1.60), *t*(587.35) = 19.42, *p* < 0.001. Table 1 contains descriptive statistics for the parent-reported SDQ.

### Measurement invariance

Table 2 contains model fit statistics for the autistic and non-autistic groups at each sweep (see Supplemental Appendix A for standardized factor loadings and covariances). With ML estimation, the chi-square test, CFI and TLI suggested inadequate fit across all groups; meanwhile, RMSEA and SRMR suggested adequate-to-good fit across all subgroups except the autistic group at age 14. With WLSMV estimation, CFI and TLI showed improved fit compared to ML estimation but remained below Hu and Bentler’s (1999) rule-of-thumb criteria. As with ML estimation, the chi-square test, CFI and TLI suggested inadequate fit across all groups, while RMSEA and SRMR suggested adequate-to-good fit across all subgroups except the autistic group at ages 14 and 17 (Supplemental Table B1). Overall, results from different estimation methods were similar and suggested inadequate fit across all groups.

**Table 2.** Fits for single-group CFA models for autistic and non-autistic groups (ML estimation).

		χ^2^ (*df*)	*p* value	CFI	TLI	RMSEA (95% CI)	SRMR
Autistic							
	Age 11	508.62 (265)	<0.001	.832	.809	.070^a^ (.059–.081)	.078^a^
	Age 14	516.22 (265)	<0.001	.842	.821	.071^a^ (.060–.082)	.084
	Age 17	541.60 (265)	<0.001	.829	.806	.074^a^ (.064–.085)	.076^a^
Non-autistic							
	Age 11	3643.53 (265)	<0.001	.852	.832	.051^b^ (.050–.053)	.043^b^
	Age 14	4871.12 (265)	<0.001	.835	.813	.060^b^ (.058–.062)	.052^b^
	Age 17	4514.68 (265)	<0.001	.850	.830	.058^b^ (.056–.059)	.050^b^

Values with no superscript indicator represent inadequate fit. CFI = comparative fit index; TLI = Tucker–Lewis index; RMSEA = root mean square error of approximation; SRMR = standardized root mean square residual; CFA = confirmatory factor analysis; ML = maximum likelihood.

a Adequate.

b Good.

Given findings of inadequate fit across all groups with the five-factor model, the possibility of improving fit by using an alternative factor structure was explored (see Supplemental Appendix C for alternative single-group model fits): the four-factor model (i.e. with the prosocial behaviour subscale removed); the three-factor model (i.e. with the conduct problems and hyperactivity/inattention items loading onto an externalizing factor, and the emotional symptoms and peer problems items loading onto an internalizing factor); the five-factor model with reverse-coded items removed. With the four-factor model, CFI and TLI showed improved fit but remained below Hu and Bentler’s (1999) criteria (Supplemental Table C1). Meanwhile, with the three-factor model, fit indices worsened, especially for the autistic group (Supplemental Table C2). Finally, removing reverse-coded items from the five-factor model improved fit indices but remained below Hu and Bentler’s (1999) criteria (Supplemental Table C3).

#### Longitudinal invariance

Table 3 contains the longitudinal analysis for the autistic group. The chi-square test, CFI and TLI suggested inadequate fit and changes in fit across all levels. Meanwhile, RMSEA and SRMR changed within acceptable criteria across all levels, with only RMSEA showing adequate fit past the configural level. Therefore, longitudinal invariance did not hold for the autistic group.

**Table 3.** Model fits for longitudinal invariance (ML estimation).

Model	Configural	Metric	Scalar	Residual
χ^2^ (*df*)	1566.44 (795)	1629.18 (835)	1736.81 (875)	1810.75 (925)
*p* value	<0.001	<0.001	<0.001	<0.001
CFI	.834	.829	.815	.810
TLI	.812	.816	.810	.815
RMSEA (95% CI)	.072^a^ (.065–.078)	.071^a^ (.065–.077)	.072^a^ (.066–.078)	.071^a^ (.065–.077)
SRMR	.076^a^	.081	.083	.087
ΔCFI	–	−.005	−.015	−.005
ΔTLI	–	+.004	−.006	+.005
ΔRMSEA	–	−.001^b^	−.001^b^	−.001^b^
ΔSRMR	–	+.005^b^	+.002^b^	+.004^b^
Model fits for longitudinal invariance (ML estimation)				
Model	Configural	Metric	Scalar	Residual
χ^2^ (*df*)	5056.28 (530)	5189.63 (550)	5271.47 (570)	6518.38 (595)
*p* value	<0.001	<0.001	<0.001	<0.001
CFI	.848	.845	.843	.802
TLI	.828	.830	.834	.800
RMSEA (95% CI)	.058^b^ (.057–.060)	.058^b^ (.056–.060)	.057^b^ (.056–.059)	.063^a^ (.061–.065)
SRMR	.049^b^	.051^b^	.051^b^	.055^b^
ΔCFI	–	−.003	−.002	−.041
ΔTLI	–	+.002	+.004	−.034
ΔRMSEA	–	.000^b^	−.001^b^	+.006^b^
ΔSRMR	–	+.002^b^	.000^b^	+.004^b^

ΔCFI, ΔTLI, ΔRMSEA and ΔSRMR represent the change in fit from a given level of invariance to the next. Values with no asterisk represent inadequate fit or change in fit. CFI = comparative fit index; TLI = Tucker–Lewis index; RMSEA = root mean square error of approximation; SRMR = standardized root mean square residual; CI = confidence interval; ML = maximum likelihood.

a Adequate.

b Good.

#### Group invariance

Table 3 contains the group analysis for 17-year-olds. With ML estimation, the chi-square test, CFI and TLI suggested inadequate fit and changes in fit across all levels, while RMSEA and SRMR showed adequate-to-good fit and changed within acceptable criteria across all levels. With WLSMV estimation, while CFI and TLI showed improved fit, results were similar to those obtained with ML estimation (Supplemental Table B2). Therefore, group invariance did not hold for 17-year-olds.

#### Differential item functioning

As a complement to MI testing, DIF was conducted to identify differences in item responses between the autistic and non-autistic groups. The Mantel–Haenszel chi-square test found that 18 of 25 items showed DIF. Five items were from the conduct problems subscale, five were from the prosocial behaviour subscale, four were from the peer problems subscale, two were from the emotional symptoms subscale and two were from the hyperactivity/inattention subscale. Table 4 contains the DIF analysis.

**Table 4.** DIF for autistic and non-autistic groups (age 17).

Item	Subscale	X^2^	Adjusted *p* value	αMH	Bias	ΔMH	Effect size
Item 1 (Being considerate of others’ feelings)	Prosocial behaviour	8347.37	<0.001***	2.46	Non-autistic	−2.11	Large
Item 4 (Sharing readily with other children)	Prosocial behaviour	1777.99	<0.001***	1.93	Non-autistic	−1.54	Large
Item 5 (Often having temper tantrums)	Conduct problems	4.78	0.042*	0.57	Autistic	1.32	Moderate
Item 7 (Generally being obedient)	Conduct problems	239.14	<0.001***	2.65	Non-autistic	−2.29	Large
Item 9 (Being helpful if someone is hurt, upset or feeling ill)	Prosocial behaviour	5787.99	<0.001***	1.49	Non-autistic	−0.93	Negligible
Item 10 (Constantly fidgeting or squirming)	Hyperactivity/inattention	28.48	<0.001***	0.20	Autistic	3.79	Large
Item 11 (Having at least one good friend)	Peer problems	517.07	<0.001***	4.13	Non-autistic	−3.33	Large
Item 12 (Often fighting with or bullying other children)	Conduct problems	15.06	<0.001***	0.35	Autistic	2.50	Large
Item 13 (Being often unhappy, down-hearted or tearful)	Emotional symptoms	4.95	0.041*	0.59	Autistic	1.26	Moderate
Item 14 (Generally being liked by other children)	Peer problems	7247.96	<0.001***	2.75	Non-autistic	−2.38	Large
Item 17 (Being kind to younger children)	Prosocial behaviour	8166.04	<0.001***	1.09	Non-autistic	−0.20	Negligible
Item 18 (Often lying or cheating)	Conduct problems	6.35	0.025*	0.58	Autistic	1.28	Moderate
Item 19 (Being picked on or bullied by other children)	Peer problems	71.03	<0.001***	0.20	Autistic	3.75	Large
Item 20 (Often volunteering to help others)	Prosocial behaviour	37.24	<0.001***	3.23	Non-autistic	−2.75	Large
Item 21 (Thinking before acting)	Hyperactivity/inattention	4.98	0.041*	3.59	Non-autistic	−3.00	Large
Item 22 (Often stealing from home, school or elsewhere)	Conduct problems	5.33	0.040*	0.44	Autistic	1.95	Large
Item 23 (Getting on better with adults than other children)	Peer problems	5.00	0.041*	0.24	Autistic	3.36	Large
Item 24 (Having many fears, being easily scared)	Emotional symptoms	4.66	0.043*	0.43	Autistic	1.97	Large

Bias represents which group favoured the item based on αMH. αMH = odds ratio; ΔMH = delta scale; DIF = differential item functioning.

* *p* < 0.05; ***p < 0.001.

Notably, DIF items included the full conduct problems and prosocial behaviour subscales, most peer problems subscale items and most reverse-coded items. Most items on the conduct problems subscale, except Item 7 (which was reverse-coded), were biased towards the autistic group (i.e. more likely to be endorsed by this group) while all items on the prosocial behaviour subscale showed non-autistic bias. Non-reverse-coded DIF items on the peer problems subscale showed autistic bias, while reverse-coded DIF items showed non-autistic bias. DIF emotional symptoms items showed autistic bias. The non-reverse-coded DIF item on the hyperactivity/inattention subscale showed autistic bias, while the reverse-coded DIF item showed non-autistic bias.

## Discussion

The current study tested MI of the parent-reported SDQ for UK-based adolescents: longitudinal invariance (autistic 11-, 14- and 17-year-olds) and group invariance (autistic and non-autistic 17-year-olds). DIF analysis was used as an exploratory analysis to identify non-invariant items. The five-factor structure showed inadequate fit across all groups, and by extension, neither longitudinal invariance across autistic 11-, 14- and 17-year-olds nor group invariance across autistic and non-autistic 17-year-olds could be established. Alternative factor structures (i.e. the four-factor and three-factor structures, as well as the five-factor structure without reverse-coded items) were explored but tended to show similarly poor or worse fit. While removing reverse-coded items was the most successful alternative factor structure in terms of improving fit, indices remained below Hu and Bentler’s (1999) rule-of-thumb criteria (see Supplemental Appendix C). Furthermore, for the group analysis, the full conduct problems and prosocial behaviour subscales, most peer problems subscale items and most reverse-coded items showed DIF, as well as two items from the emotional symptoms and hyperactivity/inattention subscales each.

Poor fit of the five-factor structure in non-autistic adolescents was inconsistent with previous findings of acceptable fit of the parent-reported (Goodman, 2001) and self-reported SDQ (Essau et al., 2012). However, more recent findings suggested that the five-factor and four-factor structures show poor fit for the self-reported SDQ in 12- to 16-year-olds, potentially due to multidimensionality of several subscales – especially hyperactivity/inattention and peer problems – based on poor fit for essential τ-equivalence (i.e. equal factor loadings of items; Kankaanpää et al., 2023). Similarly, worse fit of the three-factor structure and improved fit of the five-factor structure without reverse-coded items are consistent with previous findings in the self-reported SDQ (Essau et al., 2012), although removing items risks reducing the reliability of a short measure like the SDQ (Kankaanpää et al., 2023). Fit indices tended to be especially poor in the autistic group, suggesting that recent recommendations to revise the SDQ (Kankaanpää et al., 2023), or at least to assess the practical impact of using the SDQ in research and clinical practice, extend to its use in populations of autistic people.

To complement the main analysis, DIF analysis was used as an indicator of potential non-invariant items. DIF in the conduct problems and peer problems subscales aligned with previous findings of weak reliability for these subscales (Ribeiro Santiago et al., 2022), suggesting instrumental bias in populations of autistic people. Similarly, Staatz et al. (2021) noted that cross-loadings between subscales may be responsible for DIF items in the conduct problems, prosocial behaviour and emotional symptoms subscales, consistent with current findings. Few DIF items were found in the emotional symptoms and hyperactivity/inattention subscales; although promising given Murphy et al.’s (2018) suggestion that the parent-reported SDQ is a useful screening tool for disorders related to these subscales in populations of autistic people, poor fit of the five-factor structure in both the autistic and non-autistic groups may impact the use of the SDQ for screening purposes. Furthermore, most reverse-coded items showed DIF, consistent with van de Looij-Jansen et al.’s (2011) finding that reverse-coded items influenced factor structure due to weak correlations with other total difficulties items. They proposed that allowing reverse-coded items to cross-load onto the prosocial behaviour subscale may improve five-factor model fit. Alternatively, reformulating reverse-coded items into negatively worded items may also improve the structural validity of the SDQ (Kankaanpää et al., 2023).

Beyond identifying MI across autistic and non-autistic groups, it is arguably more important to assess the extent to which these psychometric properties have a practical impact on the use of this measure in research and clinical practice involving autistic adolescents. Despite awareness that statistical significance does not necessarily imply practical significance, this question has received relatively little consideration (M. H. C. Lai et al., 2019b). For instance, as discussed by Borsboom (2006), with sufficiently large sample sizes, all items on a given measure may show DIF, and even with moderate sample sizes, items reaching statistical significance for DIF depend on arbitrary parameters such as sample size and significance level. As such, whether DIF constitutes a threat to validity depends on the purpose for which the measure is used more so than indices of statistical significance or effect size. Similarly, the practical impact of current findings of weak structural validity and instrumental bias on the use of the SDQ depends on whether this measure is used for research or clinical practice.

In terms of practical impact on research findings, Borsboom (2006) suggested that instrumental bias caused by multidimensionality is likely to impact between- and within-group comparisons of mean scores – that is, groups may differ on a secondary latent construct that is associated with group membership but not targeted by the research. While one dimensionality of subscales was not investigated, current findings of poor five-factor model fit may be consistent with similar findings of weak structural validity and subscale multidimensionality in the self-reported SDQ (Kankaanpää et al., 2023), and thus potentially with the measurement of secondary latent constructs by items or subscales. As such, findings from research investigating relationships between SDQ scores and other variables of interest within populations of autistic adolescents, as well as comparing scores between autistic and non-autistic adolescents, may be confounded by instrumental bias. Borsboom (2006) suggested that risk of confounding is especially high where specific predictions cannot be made for the size of effects of interest, as instrumental bias may only impact findings where biasing effects are larger than the effects of interest.

Furthermore, issues with the SDQ’s validity that impact research findings are particularly significant when the measure is used for selection at the individual level (e.g. for screening purposes). While instrumental bias may cancel out at the population level, small differences in selection between a measure that is considered non-invariant, partially invariant or fully invariant may lead to misclassification in high-stakes contexts where the measure plays a major role in screening or diagnosis (M. H. C. Lai et al., 2019b). Given the need for measures that affect people’s lives directly to meet higher psychometric standards (Borsboom, 2006) and the widespread use of the SDQ to screen for mental health conditions, current findings of weak structural validity and instrumental bias in autistic and non-autistic adolescents highlight the need to revise the SDQ and to use this measure alongside less biased instruments.

### Limitations

First, unequal sample sizes between the autistic and non-autistic groups may lead to higher levels of standard error and convergence issues (Bulut, 2020). Low sample size in the autistic group may be the cause of convergence issues with WLSMV estimation at age 11 due to unequal distribution of observations across response categories (e.g. three observations for Item 21 in the 11-year-old autistic group). However, because nationally representative data were used, autism prevalence rates were similar to population estimates.

Second, fit indices may have shown inadequate fit due to the way missing data were handled. Listwise deletion under weaker Missing at Random assumptions (Liu et al., 2017) and unexplored outlier effects (van de Schoot et al., 2012) may have led to biased parameter estimates and fit indices.

Third, the possibility of item-level bias was explored through DIF analysis to complement the main analysis. However, data were not well-suited to longitudinal DIF analysis, either due to small sample size of the autistic group or the need to account for multi-level (i.e. longitudinal) data to avoid inaccurately identifying DIF items (French & Finch, 2013), a method which is not currently suitable for ordinal items (Dai et al., 2022). Attrition effects were not accounted for.

Fourth, the Mantel–Haenszel chi-square test does not differentiate between uniform and non-uniform DIF (i.e. whether DIF affects participants in different score ranges consistently). DIF analysis also does not determine fairness – that is, whether group differences in item interpretation are relevant to the constructs being tested (Martinková et al., 2017).

Finally, autism diagnosis was treated as a binary variable despite autism being a highly heterogeneous condition. M. C. Lai et al.’s (2019a) meta-analysis found substantial unexplained heterogeneity for the prevalence of mental health conditions in populations of autistic people after accounting for moderators like gender, suggesting that contributors to heterogeneity are not well accounted for; this is a fundamental limitation of the autism research literature. In addition, autism diagnosis in the MCS was determined by parent-report and not by a more reliable, formal diagnosis.

### Implications

Weak structural validity of the parent-reported SDQ, especially in autistic adolescents, as well as instrumental bias between autistic and non-autistic 17-year-olds, may have a practical impact on research and clinical practice involving autistic adolescents. Combined with recent findings of weak structural validity and subscale multidimensionality of the self-reported SDQ in non-autistic populations (Kankaanpää et al., 2023), there is increasing evidence that conclusions drawn from observed subscale and sum scores may be confounded by instrumental bias (e.g. measurement of secondary latent constructs). The widespread use of the SDQ for research on mental health conditions in populations of autistic people (e.g. measuring changes in scores to evaluate the effectiveness of interventions; Rubenstein & Bishop-Fitzpatrick, 2019) despite potential confounding by instrumental bias highlights the importance of further assessing and revising the SDQ.

Instrumental bias is particularly relevant to the use of the parent-reported SDQ to screen for mental health conditions in autistic adolescents. Even more so than for research, where biasing effects may cancel out at the population level, minimizing bias in the screening process is essential to reducing the risk of misclassifying individuals (Borsboom, 2006). Weak structural validity and instrumental bias of the SDQ should factor into clinicians’ decisions when assessing an individual’s scores (Charter & Feldt, 2001). However, given current findings of structural validity being especially weak in autistic adolescents, the use of less biased measures, either as an alternative to or alongside the SDQ, should be prioritized (Borsboom, 2006).

In addition, this instrument was designed as a broad screening tool for childhood and adolescent psychopathology, and not with populations of autistic people in mind (Simonoff et al., 2013). This further highlights the need to use the SDQ with caution (e.g. by comparing general population to autism-specific scoring methods) and alongside autism-specific measures that are well-validated for research within populations of autistic people and between autistic and non-autistic people, as well as for screening mental health conditions in autistic people. Similarly, researchers often privilege parent- and teacher-report over self-report, overlooking individual perspectives (Pellicano & Houting, 2022). This highlights the need to assess and improve the self-reported SDQ, which shows weaker validity in populations of autistic people. Growing research on the validity of the SDQ will allow for a better understanding of the unique presentation of mental health conditions in autistic people and its clinical applications (Pellicano & Houting, 2022).

### Future directions

Longitudinal and group analyses should be extended across childhood and adolescence, as well as to the self- and teacher-reported SDQ. Subscale one-dimensionality (e.g. as measured by fit for essential τ-equivalence) should be investigated in autistic adolescents to extend current findings of weak structural validity and item-level non-invariance. Investigating the practical impact of these issues on the use of the parent-reported SDQ in research and clinical practice is particularly important. In terms of impact on research findings, the robustness of effects of interest (e.g. latent score differences between autistic and non-autistic adolescents) under various levels of instrumental bias (e.g. non-invariance, partial invariance and full invariance) could be assessed (Borsboom, 2006). In terms of impact on screening outcomes, methods for assessing the impact of partial invariance (M. H. C. Lai et al., 2019b) or DIF (Gonzalez & Pelham, 2021) on diagnostic accuracy (i.e. sensitivity and specificity) could be extended for ordinal items to assess the parent-reported SDQ’s usefulness as a screening tool for mental health conditions in populations of autistic people.

Similarly, further rounds of DIF analysis and expert review of item contents are needed to determine whether DIF is a threat to validity – that is, which items may require reformulation to reduce instrumental bias. For instance, future research on the reformulation of SDQ items could follow Kankaanpää et al.’s (2023) proposed approach: focus groups and interviews with adolescents and clinicians to update our understanding of the items, followed by large-scale studies assessing the psychometric properties of the updated questionnaire. However, as noted by the authors, reformulating items in only some versions of the SDQ could complicate cross-cultural research. Alternative methods like item response theory, which provide more accurate estimates of item characteristics and latent constructs, could also be used, as well as logistic regression, which distinguishes between uniform and non-uniform DIF (Martinková et al., 2017). Further analysis, such as focus groups and follow-up interviews, is also needed to distinguish benign from adverse DIF (i.e. whether DIF reflects true differences in manifestation of latent constructs or instrumental bias) (Columbia Public Health, 2023). More advanced methods for handling missing data and outliers should be also used in future studies (Liu et al., 2017).

## Conclusion

The current study provided preliminary evidence against the structural validity of the English (UK) parent-reported SDQ across autistic and non-autistic 11-, 14- and 17-year-olds using nationally representative longitudinal data. Combined with exploratory DIF analysis, these findings suggest that future research should investigate item-level non-invariance, subscale multidimensionality and the practical impact of invalidity on research and clinical practice in populations of autistic people using more advanced methods. This research is a step towards assessing the validity of the SDQ for autism research and practice, with the aim of understanding the development of and improving screening tools for emotional and behavioural difficulties in populations of autistic people while accounting for the heterogeneity of people on the autism spectrum.

## Supplemental Material

sj-docx-1-aut-10.1177_13623613241236805 – Supplemental material for Measurement invariance of the parent-reported Strengths and Difficulties Questionnaire in autistic adolescents Supplemental material, sj-docx-1-aut-10.1177_13623613241236805 for Measurement invariance of the parent-reported Strengths and Difficulties Questionnaire in autistic adolescents by Chloe Turcan, Henry Delamain, Asher Loke, Richard Pender, Will Mandy and Rob Saunders in Autism
