# Supplementary material for: Measurement invariance of the parent-reported Strengths and Difficulties Questionnaire in autistic adolescents
Source: Autism. 2024 Mar 13;28(10):2623–36. doi: 10.1177/13623613241236805 (PMC11468119; doi:10.1177/13623613241236805)
Supplement: sj-docx-1-aut-10.1177_13623613241236805 – Supplemental material for Measurement invariance of the parent-reported Strengths and Difficulties Questionnaire in autistic adolescents [file sj-docx-1-aut-10.1177_13623613241236805.docx]

**Appendix A**

**Full Results for Single-Group CFA Models (ML Estimation)**

**Table A1**

*Parameter Estimates for Single-Group CFA Models for Autistic Group*

*Age 11 (Autistic Group Only)*

*Standardized Factor Loadings*

|  | Conduct Problems | Hyperactivity/  Inattention | Emotional Symptoms | Peer Problems | Prosocial Behaviour |
| --- | --- | --- | --- | --- | --- |
| Item 5 | .497 |  |  |  |  |
| Item 7 | -.443 |  |  |  |  |
| Item 12 | .434 |  |  |  |  |
| Item 18 | .437 |  |  |  |  |
| Item 22 | .146 |  |  |  |  |
| Item 2 |  | .553 |  |  |  |
| Item 10 |  | .512 |  |  |  |
| Item 15 |  | .556 |  |  |  |
| Item 21 |  | -.388 |  |  |  |
| Item 25 |  | -.513 |  |  |  |
| Item 3 |  |  | .318 |  |  |
| Item 8 |  |  | .612 |  |  |
| Item 13 |  |  | .490 |  |  |
| Item 16 |  |  | .414 |  |  |
| Item 24 |  |  | .525 |  |  |
| Item 6 |  |  |  | .447 |  |
| Item 11 |  |  |  | -.356 |  |
| Item 14 |  |  |  | -.351 |  |
| Item 19 |  |  |  | .385 |  |
| Item 23 |  |  |  | .425 |  |
| Item 1 |  |  |  |  | .553 |
| Item 4 |  |  |  |  | .417 |
| Item 9 |  |  |  |  | .469 |
| Item 17 |  |  |  |  | .235 |
| Item 20 |  |  |  |  | .354 |

*Note.* All factor loadings were statistically significant at p<.001.

*Factor Covariances*

|  | Conduct Problems | Hyperactivity/  Inattention | Emotional Symptoms | Peer Problems | Prosocial Behaviour |
| --- | --- | --- | --- | --- | --- |
| Conduct Problems | 1 | - | - | - | - |
| Hyperactivity/  Inattention | .612 | 1 | - | - | - |
| Emotional Symptoms | .334 | .440 | 1 | - | - |
| Peer  Problems | .358 | .418 | .636 | 1 | - |
| Prosocial Behaviour | -.636 | -.456 | -.266 | .524 | 1 |

*Note.* All covariances were statistically significant at p<.001 except emotional symptoms~~prosocial behaviour (p = .002).

*Age 14 (Autistic Group Only)*

*Standardized Factor Loadings*

|  | Conduct Problems | Hyperactivity/  Inattention | Emotional Symptoms | Peer Problems | Prosocial Behaviour |
| --- | --- | --- | --- | --- | --- |
| Item 5 | .542 |  |  |  |  |
| Item 7 | -.433 |  |  |  |  |
| Item 12 | .398 |  |  |  |  |
| Item 18 | .509 |  |  |  |  |
| Item 22 | .301 |  |  |  |  |
| Item 2 |  | .604 |  |  |  |
| Item 10 |  | .576 |  |  |  |
| Item 15 |  | .588 |  |  |  |
| Item 21 |  | -.392 |  |  |  |
| Item 25 |  | -.497 |  |  |  |
| Item 3 |  |  | .252 |  |  |
| Item 8 |  |  | .505 |  |  |
| Item 13 |  |  | .429 |  |  |
| Item 16 |  |  | .479 |  |  |
| Item 24 |  |  | .641 |  |  |
| Item 6 |  |  |  | .449 |  |
| Item 11 |  |  |  | -.455 |  |
| Item 14 |  |  |  | -.469 |  |
| Item 19 |  |  |  | .442 |  |
| Item 23 |  |  |  | .297 |  |
| Item 1 |  |  |  |  | .447 |
| Item 4 |  |  |  |  | .434 |
| Item 9 |  |  |  |  | .492 |
| Item 17 |  |  |  |  | .263 |
| Item 20 |  |  |  |  | .398 |

*Note.* All factor loadings were statistically significant at p<.001.

*Factor Covariances*

|  | Conduct Problems | Hyperactivity/  Inattention | Emotional Symptoms | Peer Problems | Prosocial Behaviour |
| --- | --- | --- | --- | --- | --- |
| Conduct Problems | 1 | - | - | - | - |
| Hyperactivity/  Inattention | .615 | 1 | - | - | - |
| Emotional Symptoms | .191 | .335 | 1 | - | - |
| Peer  Problems | .396 | .396 | .551 | 1 | - |
| Prosocial Behaviour | -.571 | -.375 | -.287 | -.428 | 1 |

*Note.* All covariances were statistically significant at p<.001 except conduct problems~~emotional symptoms (p=.027).

*Age 17 (Autistic Group Only)*

*Standardized Factor Loadings*

|  | Conduct Problems | Hyperactivity/  Inattention | Emotional Symptoms | Peer Problems | Prosocial Behaviour |
| --- | --- | --- | --- | --- | --- |
| Item 5 | .431 |  |  |  |  |
| Item 7 | -.393 |  |  |  |  |
| Item 12 | .284 |  |  |  |  |
| Item 18 | .449 |  |  |  |  |
| Item 22 | .296 |  |  |  |  |
| Item 2 |  | .628 |  |  |  |
| Item 10 |  | .615 |  |  |  |
| Item 15 |  | .606 |  |  |  |
| Item 21 |  | -.418 |  |  |  |
| Item 25 |  | -.499 |  |  |  |
| Item 3 |  |  | .339 |  |  |
| Item 8 |  |  | .619 |  |  |
| Item 13 |  |  | .489 |  |  |
| Item 16 |  |  | .517 |  |  |
| Item 24 |  |  | .565 |  |  |
| Item 6 |  |  |  | .514 |  |
| Item 11 |  |  |  | -.506 |  |
| Item 14 |  |  |  | -.450 |  |
| Item 19 |  |  |  | .367 |  |
| Item 23 |  |  |  | .173 |  |
| Item 1 |  |  |  |  | .415 |
| Item 4 |  |  |  |  | .419 |
| Item 9 |  |  |  |  | .416 |
| Item 17 |  |  |  |  | .296 |
| Item 20 |  |  |  |  | .399 |

*Note.* All factor loadings were statistically significant at p<.001 except item 23 onto the prosocial behaviour subscale (p=.002).

*Factor Covariances*

|  | Conduct Problems | Hyperactivity/  Inattention | Emotional Symptoms | Peer Problems | Prosocial Behaviour |
| --- | --- | --- | --- | --- | --- |
| Conduct Problems | 1 | - | - | - | - |
| Hyperactivity/  Inattention | .584 | 1 | - | - | - |
| Emotional Symptoms | .294 | .476 | 1 | - | - |
| Peer  Problems | .265 | .299 | .465 | 1 | - |
| Prosocial Behaviour | -.546 | -.398 | -.378 | -.507 | 1 |

*Note.* All covariances were statistically significant at p<.001 except conduct problems~~emotional symptoms (p=.001) and conduct problems~~peer problems (p=.003).

**Table A2**

*Parameter Estimates for Single-Group CFA Models for Non-Autistic Group*

*Age 11 (Non-Autistic Group Only)*

*Standardized Factor Loadings*

|  | Conduct Problems | Hyperactivity/  Inattention | Emotional Symptoms | Peer Problems | Prosocial Behaviour |
| --- | --- | --- | --- | --- | --- |
| Item 5 | .401 |  |  |  |  |
| Item 7 | -.309 |  |  |  |  |
| Item 12 | .100 |  |  |  |  |
| Item 18 | .186 |  |  |  |  |
| Item 22 | .042 |  |  |  |  |
| Item 2 |  | .410 |  |  |  |
| Item 10 |  | .344 |  |  |  |
| Item 15 |  | .495 |  |  |  |
| Item 21 |  | -.319 |  |  |  |
| Item 25 |  | -.414 |  |  |  |
| Item 3 |  |  | .229 |  |  |
| Item 8 |  |  | .401 |  |  |
| Item 13 |  |  | .232 |  |  |
| Item 16 |  |  | .303 |  |  |
| Item 24 |  |  | .345 |  |  |
| Item 6 |  |  |  | .222 |  |
| Item 11 |  |  |  | -.132 |  |
| Item 14 |  |  |  | -.189 |  |
| Item 19 |  |  |  | .304 |  |
| Item 23 |  |  |  | .253 |  |
| Item 1 |  |  |  |  | .255 |
| Item 4 |  |  |  |  | .237 |
| Item 9 |  |  |  |  | .207 |
| Item 17 |  |  |  |  | .140 |
| Item 20 |  |  |  |  | .238 |

*Note.* All covariances were statistically significant at p<.001.

*Factor Covariances*

|  | Conduct Problems | Hyperactivity/  Inattention | Emotional Symptoms | Peer Problems | Prosocial Behaviour |
| --- | --- | --- | --- | --- | --- |
| Conduct Problems | 1 | - | - | - | - |
| Hyperactivity/  Inattention | .711 | 1 | - | - | - |
| Emotional Symptoms | .445 | .390 | 1 | - | - |
| Peer  Problems | .478 | .421 | .644 | 1 | - |
| Prosocial Behaviour | -.599 | -.363 | -.148 | -.294 | 1 |

*Note.* All covariances were statistically significant at p<.001.

*Age 14 (Non-Autistic Group Only)*

*Standardized Factor Loadings*

|  | Conduct Problems | Hyperactivity/  Inattention | Emotional Symptoms | Peer Problems | Prosocial Behaviour |
| --- | --- | --- | --- | --- | --- |
| Item 5 | .403 |  |  |  |  |
| Item 7 | -.326 |  |  |  |  |
| Item 12 | .105 |  |  |  |  |
| Item 18 | .226 |  |  |  |  |
| Item 22 | .088 |  |  |  |  |
| Item 2 |  | .349 |  |  |  |
| Item 10 |  | .296 |  |  |  |
| Item 15 |  | .512 |  |  |  |
| Item 21 |  | -.357 |  |  |  |
| Item 25 |  | -.441 |  |  |  |
| Item 3 |  |  | .253 |  |  |
| Item 8 |  |  | .434 |  |  |
| Item 13 |  |  | .295 |  |  |
| Item 16 |  |  | .364 |  |  |
| Item 24 |  |  | .335 |  |  |
| Item 6 |  |  |  | .272 |  |
| Item 11 |  |  |  | -.159 |  |
| Item 14 |  |  |  | -.246 |  |
| Item 19 |  |  |  | .278 |  |
| Item 23 |  |  |  | .253 |  |
| Item 1 |  |  |  |  | .343 |
| Item 4 |  |  |  |  | .299 |
| Item 9 |  |  |  |  | .271 |
| Item 17 |  |  |  |  | .204 |
| Item 20 |  |  |  |  | .343 |

*Note.* All covariances were statistically significant at p<.001.

*Factor Covariances*

|  | Conduct Problems | Hyperactivity/  Inattention | Emotional Symptoms | Peer Problems | Prosocial Behaviour |
| --- | --- | --- | --- | --- | --- |
| Conduct Problems | 1 | - | - | - | - |
| Hyperactivity/  Inattention | .697 | 1 | - | - | - |
| Emotional Symptoms | .408 | .373 | 1 | - | - |
| Peer  Problems | .540 | .428 | .643 | 1 | - |
| Prosocial Behaviour | -.653 | -.442 | -.173 | .407 | 1 |

*Note.* All covariances were statistically significant at p<.001.

*Age 17 (Non-Autistic Group Only)*

*Standardized Factor Loadings*

|  | Conduct Problems | Hyperactivity/  Inattention | Emotional Symptoms | Peer Problems | Prosocial Behaviour |
| --- | --- | --- | --- | --- | --- |
| Item 5 | .369 |  |  |  |  |
| Item 7 | -.327 |  |  |  |  |
| Item 12 | .072 |  |  |  |  |
| Item 18 | .197 |  |  |  |  |
| Item 22 | .079 |  |  |  |  |
| Item 2 |  | .296 |  |  |  |
| Item 10 |  | .256 |  |  |  |
| Item 15 |  | .481 |  |  |  |
| Item 21 |  | -.346 |  |  |  |
| Item 25 |  | -.445 |  |  |  |
| Item 3 |  |  | .289 |  |  |
| Item 8 |  |  | .490 |  |  |
| Item 13 |  |  | .338 |  |  |
| Item 16 |  |  | .398 |  |  |
| Item 24 |  |  | .370 |  |  |
| Item 6 |  |  |  | .328 |  |
| Item 11 |  |  |  | -.184 |  |
| Item 14 |  |  |  | -.241 |  |
| Item 19 |  |  |  | .195 |  |
| Item 23 |  |  |  | .272 |  |
| Item 1 |  |  |  |  | .325 |
| Item 4 |  |  |  |  | .284 |
| Item 9 |  |  |  |  | .276 |
| Item 17 |  |  |  |  | .180 |
| Item 20 |  |  |  |  | .346 |

*Note.* All covariances were statistically significant at p<.001.

*Factor Covariances*

|  | Conduct Problems | Hyperactivity/  Inattention | Emotional Symptoms | Peer Problems | Prosocial Behaviour |
| --- | --- | --- | --- | --- | --- |
| Conduct Problems | 1 | - | - | - | - |
| Hyperactivity/  Inattention | .721 | 1 | - | - | - |
| Emotional Symptoms | .404 | .438 | 1 | - | - |
| Peer  Problems | .439 | .441 | .647 | 1 | - |
| Prosocial Behaviour | -.686 | -.441 | -.201 | -.447 | 1 |

*Note.* All covariances were statistically significant at p<.001.

**Appendix B**

**Measurement Invariance Testing with WLSMV Estimation**

**Table B1**

*Fits for Single-Group CFA Models for Autistic and Non-Autistic Groups (WLSMV Estimation)*

|  |  | χ2 (*df*) | *p*-value | CFI | TLI | RMSEA (95% CI) | SRMR |
| --- | --- | --- | --- | --- | --- | --- | --- |
| Autistic |  |  |  |  |  |  |  |
|  | **Age 11** | NA | NA | NA | NA | NA | NA |
|  | **Age 14** | 514.41 (265) | < .001 | .910 | .898 | .071* (.060-.082) | .104 |
|  | **Age 17** | 418.34 (265) | < .001 | .943 | .936 | .055** (.043-.067) | .094 |
| Non-autistic |  |  |  |  |  |  |  |
|  | **Age 11** | 3272.92 (265) | < .001 | .917 | .906 | .048** (.047-.050) | .067* |
|  | **Age 14** | 4776.07 (265) | < .001 | .895 | .881 | .059** (.058-.061) | .075* |
|  | **Age 17** | 4140.80 (265) | < .001 | .913 | .901 | .055** (.053-.057) | .074* |

*Note.* No asterisk = inadequate fit; * = adequate; ** = good. CFI = comparative fit index; TLI = Tucker-Lewis index; RMSEA = root mean square error of approximation; SRMR = standardized root mean square residual. Scaled chi-squared test results and fit indices were reported for WLSMV estimation.

**Table B2**

*Model Fits for Group Invariance (WLSMV Estimation)*

| Model | Configural | Metric | Scalar | Residual |
| --- | --- | --- | --- | --- |
| χ2 (*df*) | 3828.14 (530) | 3368.46 (550) | 3707.97 (570) | 3707.97 (570) |
| *p*-value | < .001 | < .001 | < .001 | < .001 |
| CFI | .920 | .932 | .924 | .924 |
| TLI | .910 | .926 | .920 | .920 |
| RMSEA (95% CI) | .050** (.048-.052) | .045** (.043-.047) | .047** (.045-.049) | .047** (.045-.049) |
| SRMR | .075* | .075* | .075* | .075* |
| ΔCFI | - | +.012 | -.008 | .000** |
| ΔTLI | - | +.016 | -.006 | .000** |
| ΔRMSEA | - | -.005** | +.002** | .000** |
| ΔSRMR | - | .000** | .000** | .000** |

*Note.* ΔCFI, ΔTLI, ΔRMSEA, and ΔSRMR represent the change in fit from a given level of invariance to the next. No asterisk = inadequate fit or change in fit; * = adequate; ** = good. CFI = comparative fit index; TLI = Tucker-Lewis index; RMSEA = root mean square error of approximation; SRMR = standardized root mean square residual. Scaled chi-squared test results and fit indices were reported for WLSMV estimation.

**Appendix C**

**Model Fits for Alternative Factor Structures (ML Estimation)**

**Table C1**

*Fits for Four-Factor Model for Autistic and Non-Autistic Groups*

|  |  | χ2 (*df*) | *p*-value | CFI | TLI | RMSEA (90% CI) | SRMR |
| --- | --- | --- | --- | --- | --- | --- | --- |
| Autistic |  |  |  |  |  |  |  |
|  | **Age 11** | 328.44 (164) | < .001 | .847 | .823 | .073* (.059-.086) | .075* |
|  | **Age 14** | 342.04 (164) | < .001 | .858 | .836 | .076* (.062-.089) | .082 |
|  | **Age 17** | 360.80 (164) | < .001 | .847 | .822 | .080* (.066-.093) | .076* |
| Non-autistic |  |  |  |  |  |  |  |
|  | **Age 11** | 2664.56 (164) | < .001 | .867 | .846 | .056** (.054-.058) | .041** |
|  | **Age 14** | 3433.86 (164) | < .001 | .842 | .817 | .064* (.062-.066) | .047** |
|  | **Age 17** | 3190.49 (164) | < .001 | .857 | .835 | .062* (.060-.064) | .046** |

*Note.* No asterisk = inadequate fit; * = adequate; ** = good. CFI = comparative fit index; TLI = Tucker-Lewis index; RMSEA = root mean square error of approximation; SRMR = standardized root mean square residual.

**Table C2**

*Fits for Three-Factor Models for Autistic and Non-Autistic Groups*

|  |  | χ2 (*df*) | *p*-value | CFI | TLI | RMSEA (95% CI) | SRMR |
| --- | --- | --- | --- | --- | --- | --- | --- |
| Autistic |  |  |  |  |  |  |  |
|  | **Age 11** | 645.10 (272) | < .001 | .742 | .715 | .085 (.075-.095) | .089 |
|  | **Age 14** | 704.95 (272) | < .001 | .728 | .700 | .092 (.082-.102) | .095 |
|  | **Age 17** | 745.16 (272) | < .001 | .707 | .677 | .096 (.086-.106) | .092 |
| Non-autistic |  |  |  |  |  |  |  |
|  | **Age 11** | 5115.47 (272) | < .001 | .787 | .765 | .061* (.059-.062) | .053** |
|  | **Age 14** | 6676.23 (272) | < .001 | .770 | .746 | .070* (.068-.072) | .063* |
|  | **Age 17** | 6147.91 (272) | < .001 | .792 | .770 | .067* (.065-.069) | .062* |

*Note.* No asterisk = inadequate fit; * = adequate; ** = good. CFI = comparative fit index; TLI = Tucker-Lewis index; RMSEA = root mean square error of approximation; SRMR = standardized root mean square residual.

**Table C3**

*Fits for Five-Factor Models Without Reverse-Coded Items for Autistic and Non-Autistic Groups*

|  |  | χ2 (*df*) | *p*-value | CFI | TLI | RMSEA (95% CI) | SRMR |
| --- | --- | --- | --- | --- | --- | --- | --- |
| Autistic |  |  |  |  |  |  |  |
|  | **Age 11** | 282.07 (160) | < .001 | .880 | .858 | .064* (.049-.078) | .073* |
|  | **Age 14** | 260.80 (160) | < .001 | .910 | .893 | .058** (.042-.072) | .075* |
|  | **Age 17** | 282.26 (160) | < .001 | .894 | .874 | .064* (.049-.078) | .075* |
| Non-autistic |  |  |  |  |  |  |  |
|  | **Age 11** | 1330.75 (160) | < .001 | .924 | .910 | .039** (.037-.041) | .033** |
|  | **Age 14** | 2137.12 (160) | < .001 | .899 | .880 | .051** (.048-.053) | .045** |
|  | **Age 17** | 1939.87 (160) | < .001 | .911 | .895 | .048** (.046-.050) | .040** |

*Note.* No asterisk = inadequate fit; * = adequate; ** = good. CFI = comparative fit index; TLI = Tucker-Lewis index; RMSEA = root mean square error of approximation; SRMR = standardized root mean square residual.
